# The teaching and learning environment of a primary care medical student clinical attachment (“Famulatur”) – a qualitative study on experiences of students and primary care physicians in Germany

**DOI:** 10.3205/zma001236

**Published:** 2019-05-16

**Authors:** Kirsten Gottlob, Stefanie Joos, Hannah Haumann

**Affiliations:** 1 University Hospital Tübingen, Institute for General Practice and Interprofessional Care, Tübingen, Germany

**Keywords:** primary care, general practice, medical education, clinical attachment, famulatur

## Abstract

**Aim:** Following changes in licensing regulations for doctors (“Approbationsordnung”) in 2012, a 4-week clinical attachment (“Famulatur”) in primary care is now mandatory for all medical students in Germany. To date, it has not been studied how the Famulatur in primary care is perceived by the learner or the teacher. The aim of this study was to explore the experiences of both medical students and primary care physicians (PCPs) with regard to the teaching and learning situation in the Famulatur in primary care.

**Methods: **A qualitative analysis of semi-structured interviews with 12 students from the medical faculty in Tübingen, Germany, and 17 PCPs from this region, was performed. Interview material was analyzed following content analysis according to Mayring.

**Results:** In addition to considering the variety of tasks expected of the students and the optimal time for the Famulatur during the medical curriculum, the main themes of the interviews were the strengths, weaknesses and suggestions for improvement of the Famulatur. The Famulatur was predominantly perceived positively, although it being obligatory was criticized. In particular, the 1:1 supervision and the extended duration (compared to the first curricular primary care placement (“Blockpraktikum”)) were positively evaluated. PCPs and students were critical of the lack of a learning and educational Famulatur framework, which would have enabled earlier orientation and alignment of each party.

**Conclusion:** The Famulatur offers good learning opportunities for medical students and provides an insight into primary care, which is typically seen positively; it appears to heighten enthusiasm for primary care within budding doctors. Even if the obligation should cease in “The Master Plan for Medical Studies 2020” (Masterplan Medizinstudium 2020), it would be beneficial to optimize the primary care Famulatur; the development of a student logbook and learning objectives could be helpful, for example in the form of portfolios.

## Zusammenfassung

**Zielsetzung: **Seit der Änderung der Approbationsordnung für Ärzte im Jahr 2012 ist eine vierwöchige Famulatur im hausärztlichen Bereich abzuleisten. Bisher ist kaum bekannt, wie diese Lehr- bzw. Lernsituation von den Beteiligten wahrgenommen wird. Ziel dieser Untersuchung ist es, Erfahrungen von Medizinstudierenden und Hausärzten auf die Lehr- und Lernsituation in der Famulatur in der hausärztlichen Versorgung zu explorieren.

**Methoden: **Es erfolgte eine qualitative Analyse mit Leitfaden-gestützten Einzelinterviews mit 12 Studierenden der Universität Tübingen sowie 17 Hausärzten aus dieser Region. Die Auswertung erfolgte analog der qualitativen Inhaltsanalyse nach Mayring.

**Ergebnisse: **Neben dem Aufgabenspektrum und dem besten Zeitpunkt der Famulatur wurden die Teilnehmer vor allem zu Stärken, Schwächen sowie Verbesserungsvorschlägen der Famulatur befragt. Die Famulatur wurde überwiegend positiv wahrgenommen, wobei der Pflichtcharakter kritisiert wurde. Insbesondere positiv bewertet wurde die 1:1-Betreuung und der im Vergleich zum Blockpraktikum längere Zeitraum. Hausärzte und Studierende beanstandeten das Fehlen von Lernzielen zur Orientierung und Strukturierung der Famulatur.

**Schlussfolgerung: **Die Famulatur in der hausärztlichen Versorgung bietet insgesamt gute Lernmöglichkeiten für Medizinstudierende und die Möglichkeit, für den Hausarztberuf zu begeistern. Auch wenn die Verpflichtung im Zuge des Masterplans 2020 entfallen sollte, wäre es wünschenswert Möglichkeiten zur Verbesserung der Famulatur Allgemeinmedizin zu erarbeiten. Die Entwicklung eines Logbuchs für Studierende sowie eines Lernzielkatalogs stellen hilfreiche Ideen dar.

## 1. Introduction

The clinical attachment (“Famulatur”) is an important practical part of medical school in Germany. An amendment of the licensing regulations for doctors (“Approbationsordnung für Ärzte, ÄAppo”) in 2012 stipulated that a one-month Famulatur must be carried out in primary care, i.e. with a general practitioner (GP), an internist working in primary care or a paediatrician in private practice. In 2002, a primary care placement (“Blockpraktikum”) was added to the curriculum. Since 2012 this compulsory Blockpraktikum has to be completed by all German medical students for a minimum period of two weeks. The aim of these changes to the licensing regulations was to inspire future physicians to take up a career in primary care [1]. It is known that early exposure to primary care during medical school, and prolonged practical training in primary care are positive influencing factors for a later career as a primary care phyisican (PCP) [2], [3], [4], [5]. A study from the USA found that after a 3-week placement in primary care, students showed an increased interest in primary care [6]. 

Apart from the time frame, the ÄAppO does not define any objectives of the Famulatur; in particular, there are no teaching or learning goals. In its “Recommendations for the future development of medical schools in Germany” of July 2014, the German Science Council (Wissenschaftsrat) also overlooks the topic of the Famulatur [7]. Each medical faculty has no influence on the choice of practice by the students or governance of the practice. It can therefore be assumed that there are structural and qualitative differences between such practices. In order to counter this, in 2013 a “Joint Working Group on the Famulatur in primary care” was formed comprising of The German College of General Practitioners and Family Physicians (Deutsche Gesellschaft für Allgemeinmedizin und Familienmedizin, DEGAM), The Association of Family Physicians and General Practitioners (Deutscher Hausärzteverband, HÄV) and The German Medical Student Association (Bundesvertretung der Medizinstudierenden in Deutschland, BVMD), amongst others, and a “learning and teaching aid for the Famulatur in primary care” was published to be used as a guideline by students and teachers [8]. 

A survey of PCPs in Lower Saxony showed that typical PCP skills such as history-taking and physical examination were, in particular, considered to be relevant learning content [9]. Since 2011, The Institute for General Practice and Health Service Research at the Technical University (TU) in Munich has been offering an multi-disciplinary concept for the Famulatur in primary care. The Munich model, FAMULATUM, offers clinical mentors, e-learning and goal-oriented learning. Its aim is to improve the Famulatur in primary care and increase its attractiveness [10]. In other European and non-European countries, the concept of Famulatur is less established, and curricular clinical placements dominate [11], [12]. In Austria, for example, a five-week compulsory placement in primary care was introduced at the Medical University of Graz in 2007, which was very well evaluated by both teaching practices and students [13]. In 2014, the placement in primary care was integrated into the final practical year [14].

In other medical specialties, the qualitative experiences of the Famulatur are also underinvestigated. A study from orthopedics and trauma surgery shows that there are large differences within the Famulatur regarding “practical relevance of student activities, and structure [...] and achievement of learning goals” [15]. A qualitative study in surgery also criticized the “lack of defined training goals for practical skills” and a strong “dependence on the motivation and interest of individual teachers” [16].

Little is known about the experience of the Famulatur in primary care by students and the PCPs who offer it. At the Medical Faculty of Tübingen, the Famulatur in primary care is usually completed between the 5^th^ and the 9^th^ semester and, therefore, before the Blockpraktikum, which takes place in the 10^th^ semester and shortly before the practical year. The Blockpraktikum is a mandatory two week clinical placement within teaching practices linked to the medical faculty of Tübingen. All primary care practices in Baden-Württemberg are entitled to a grant for the Famulatur (160 €/month, max. 2 months per student), which is to be passed on to the student via the Association of Physicians’ Statutory Health Insurance (Kassenärztliche Vereinigung, KV). It is to date unexplored as to whether and how students prepare for their Famulatur in primary care and how the practices for the Famulatur are chosen. The following explorative study considers the teaching and learning experiences of both the students and PCPs during the primary care Famulatur of the Medical Faculty Tübingen. In particular, the aspects of acceptance, implementation and the relevance of prior Famulatur experience within primary care will be discussed. 

## 2. Methods

This was an explorative study using a qualitative approach. We conducted semi-structured interviews with PCPs and medical students. The interviews were one-on-one to avoid mutual influence between the participants. PCPs were recruited via the medical directory of the Association of Physicians’ Statutory Health Insurance (Kassenärztliche Vereinigung, KV) from Tübingen, Reutlingen and Rottenburg (south-west Germany). A total of 76 PCPs were randomly selected from the list and contacted in writing during two recruitment periods (first recruitment period every fourth physician from the medical directory, second recruitment period every second physician from the medical directory apart from those who had already been contacted; response rate 22%). 17 PCPs agreed to participate. The interviews were conducted by telephone. 12 medical students of the Medical Faculty of Tübingen, who had agreed to participate voluntarily following an e-mail call to all third, fourth and fifth year medical students, were interviewed. Each had completed their Famulatur in primary care. Since the exact number of medical students contacted was not known, no response rate could be determined. The interviews were subsequently transcribed. The content analysis was carried out by a team of two researchers, a medical student and a PCP. As per Mayring, content analysis was performed and main and subcategories derived from the text material deductively and inductively [17]. Finally, the text material was compared: first the texts within one category were compared with each other and then a comparison between the different categories was carried out. This study received approval from the ethics commission of the Medical Faculty of Tübingen (file number 694/2015BO2).

## 3. Results

17 PCPs and 12 students took part in the interviews. Table 1 (Tab. 1) shows the sociodemographic data of the physicians and students interviewed plus the characteristics of the practices the participating PCPs worked at. PCPs and medical students answered questions, amongst others, on the following main categories that were derived deductively from the interviews: “Associations with Famulatur in primary care”, “Strengths of the Famulatur in primary care”, “Weaknesses of the Famulatur in primary care” and “Suggestions for improvement”. In addition, the differences between the curricular Blockpraktikum in primary care and the Famulatur in primary care was part of the questions asked of the surveyed PCPs who were also faculty teaching physicians. The detailed results are presented in attachment 1 (Content analysis: main categories and selected subcategories from interviews with primary care physicians) and attachment 2 (Content analysis: main categories and selected subcategories from interviews with medical students). 

In the interviews, the following positive aspects of the Famulatur in primary care were mentioned: Students enjoyed the wide range of reasons for seeking treatment and the close patient contact, as well as the complex clinical pictures that challenged them intellectually. The 1:1 supervision was evaluated by the students as a clear strength of the Famulatur. Other students pointed out that “*they could take care of their own patients”*. The latter was not mentioned by the PCPs. The PCPs, on the other hand, stated that they could spark students’ interest in primary care. A few students were critical of their role during the Famulatur, which was sometimes too passive or that there were too few opportunities to work independently. Occasionally, students did not perceive primary care as a medical specialty of its own and regarded the Famulatur as an obligation only. An aspect emphasized by students and PCPs as a limiting factor of the Famulatur was the consultation time pressures. In addition, some PCPs reported that it was difficult for students to establish regular patient contact because patients preferred to see their PCP. Some PCPs highlighted that students underestimated the speciality of primary care. 

The students suggested the need for a framework as the main area of improvement: specifically, guidelines for each party in order to better assign tasks and to provide an overview of learning objectives. The PCPs mentioned how useful financial compensation for the teaching time would be and would further incentivise participation. 

A further category derived from the interviews was the distinction between the curricular Blockpraktikum and the Famulatur in primary care (“Famulatur”; not shown in attachment 1 and attachment 2 ). The main observation made comparing the two was that the students often had less clinical knowledge and would work less independently during their Famulatur [Note: The curricular Blockpraktikum at the Medical Faculty of Tübingen is at the end of the clinical training in the 10^th^ semester]:* “So the students during the curricular Blockpraktikum are further on in their training, and then they can do more. You can then discuss complex clinical pictures or something like that with them. [...]”* {HA16} 

It was emphasized that the faculty could garner interest in primary care earlier in the medical curriculum, for example during the Famulatur, and individually tailored programs would be better: *“The disadvantage of the curricular Blockpraktikum is that these are students who nearly all have already decided on their choice of medical specialty and then they clearly signal that they are only doing [the Famulatur] because they have to and have no interest in primary care”.* {HA9} It was positively noted that the Famulatur was a longer duration than the two-week curricular Blockpraktikum. Students during the curricular Blockpraktikum were often very focussed on how they performed, whilst the students during the Famulatur seemed more relaxed and less conscious as to how they were performing. With respect to the organization and planning, it was noted that students could not be accepted for the Famulatur in addition to the Blockpraktikum because of the additional workload for the PCP. 

## 4. Discussion

The aim of this study was to explore the degree of acceptance towards, the implementation of and the experiences garnered from the Famulatur in primary care.

From the interview material it was clear that interest in primary care amongst medical students can be influenced by their Famulatur and their attitude towards the speciality can be changed (see, for example, categories “Associations with the term Famulatur in primary care” and “Strengths of the Famulatur in primary care”, attachment 2 ). This phenomenon is further supported within international literature. However, the concept of a Famulatur in primary care is more specific to Germany; studies concerning clinical attachments more analogous to the curricular Blockpraktikum predominate in the literature. A study by Morrison et al. showed that after completing a placement in primary care at the University of Glasgow (4 weeks duration, last year of medical school) students showed a more positive attitude towards the subject, but this interest was not sustained by the end of their training [18]. Evaluation of the British curriculum (which incorporates repeated training in primary care) and studies from Baden-Württemberg also showed a positive influence on the attitude of students towards primary care [19], [20], [21]. Early contact with the field of primary care during medical school and longer periods of practical training were found in several studies to be positive influencing factors affecting the later decision to train and work in primary care [3], [5]. Evaluations of the curricular Blockpraktikum in primary care in Germany also show that positive learning experiences increase motivation for primary care [22], [23]. Furthermore, it is known from a qualitative study using focus groups that the attitudes of medical students towards primary care before completing a primary care placement are typically based on subjective prejudices or false information [24]. Indeed, one student within our study remarked that primary care was not a specialty of its own. The Famulatur in primary care, therefore, offers the opportunity to reduce prejudices and promote interest in primary care. 

Almost all students interviewed had not had contact with primary care before their Famulatur. PCPs and students rated the 1:1 supervision during the Famulatur as very positive. Although PCPs feared that students underestimated the complexity of their specialty, it was found that students reflected on the core elements of primary care and rated them as demanding. This, in turn, corresponded to the PCPs' expectations of the goals for the Famulatur and their motivation to supervise students. It can be assumed that the intensive 1:1 supervision during the Famulatur gives students a deeper insight into the work of a PCP [10]. This exclusive learning situation offers the opportunity to expand on the individual’s knowledge and the learning goals derived from it. 

Each party suggested an introductory and summary discussion about the Famulatur, and to catalogue learning objectives as ways to improve the experience and to aid integration of the students. Recommendations for the Famulatur of the standing committee in primary care of The German Association for Medical Education (Gesellschaft für Medizinische Ausbildung, GMA) also suggest introductory talks and regular feedback to the students from the supervising physicians. The discussions serve to make the Famulatur more bespoke to the individual’s interests and prior knowledge [25]. A learning objectives catalogue could further support this process. In the FAMULATUM model of the TU Munich, a learning objectives catalogue is tailored to the student’s level of knowledge in cooperation with a mentor [10]. Surveys of the students showed that the areas “common diseases” and “communication skills” can be taught well in most PCP practices [26]. In addition, the Famulatur in primary care offers the opportunity to become familiar with a diagnostic process based on signs and symptoms and the caring for patients without established diagnoses. These are precisely the learning points that distinguish the Famulatur in primary care from others. 

Students' criticism that there was little autonomy during the Famulatur is multifactorial. In addition to the level of medical knowledge and commitment of the students, the willingness of the patients and the interpersonal dynamic with the PCPs (such as their didactic style or otherwise) also play a role. In addition, it is important that the students are integrated into daily routines in the practice. With regard to this, the PCPs noted that the time pressure in the practices could conflict with the supervision of students. This challenge is also described in an Australian study and a survey from Austria suggests the presence of students may lead to longer consultation times [27], [28].

The main difference between the Famulatur and the Blockpraktikum in primary care was the flexibility of the Famulatur. It can be assumed that this freedom could be more orientated if teachers and learners had a framework e.g. learning objectives. Of note, many PCPs have already received clinical teacher training but that such training is not a prerequisite to offer a Famulatur. 

This study is one of few concerning Famulatur and one of fewer concerning the Famulatur specifically in primary care. Our qualitative data may be vulnerable to selection bias given the high number of teaching physicians among the respondents.

The data was collected from students of only one medical faculty and PCPs of only one region and using semi-structured interviews; this can only enable limited conclusions to be drawn. The specific regulations of the curriculum at the Medical Faculty of Tübingen (e.g. Blockpraktikum usually after the Famulatur) also limit the pool from which to draw interviewees as well as the varying teaching experience of the physicians of the Institute for General Practice and Interprofessional Care in Tübingen. Only limited sociodemographic characteristics were surveyed, such that a more detailed analysis of the results (e.g. possible links between the perception of the Famulatur and the age of the PCPs) was not possible. 

## 5. Conclusions

At a time when the number of PCPs has been declining for years, the Famulatur in primary care offers students an opportunity to become acquainted with the working methods and characteristics of primary care [22]. Both students and PCPs draw a wide range of experiences from the Famulatur. Within the framework of the “The Master Plan for Medical Studies 2020” (Masterplan Medizinstudium 2020), changes are planned for the last year of medical school, the Practical Year (PJ), amongst others. A compulsory time in the outpatient or primary care setting is to be established. In addition, there is planned to be a mandatory examination on primary care in the medical undergraduate final exam. At the same time, the obligatory curricular clinical attachment in primary care is to be removed. Nevertheless, it will still be possible to complete a four-week Famulatur at a PCP. For this reason, opportunities for improvement of the Famulatur in primary care should not be ignored. For example, the interviewees mentioned the creation of a framework with learning goals for PCPs and students. Of note, Famulatur checklists for students and supervisors have already been developed within the DEGAM standing committee Famulatur. These are freely available [https://degam-famulaturboerse.de/]. Such a proposed improvement of the Famulatur in primary care could serve as inspiration for other specialties. 

## Acknowledgement

We would like to thank all students of the Medical Faculty Tübingen as well as all PCPs for their participation in the study.

## Competing interests

The authors declare that they have no competing interests. 

## Supplementary Material

Content analysis: main categories and selected subcategories from interviews with family physicians (PCP: primary care physician)

Main and selected subcategories of content from student interviews (PCP: primary care physician)

## Figures and Tables

**Table 1:**
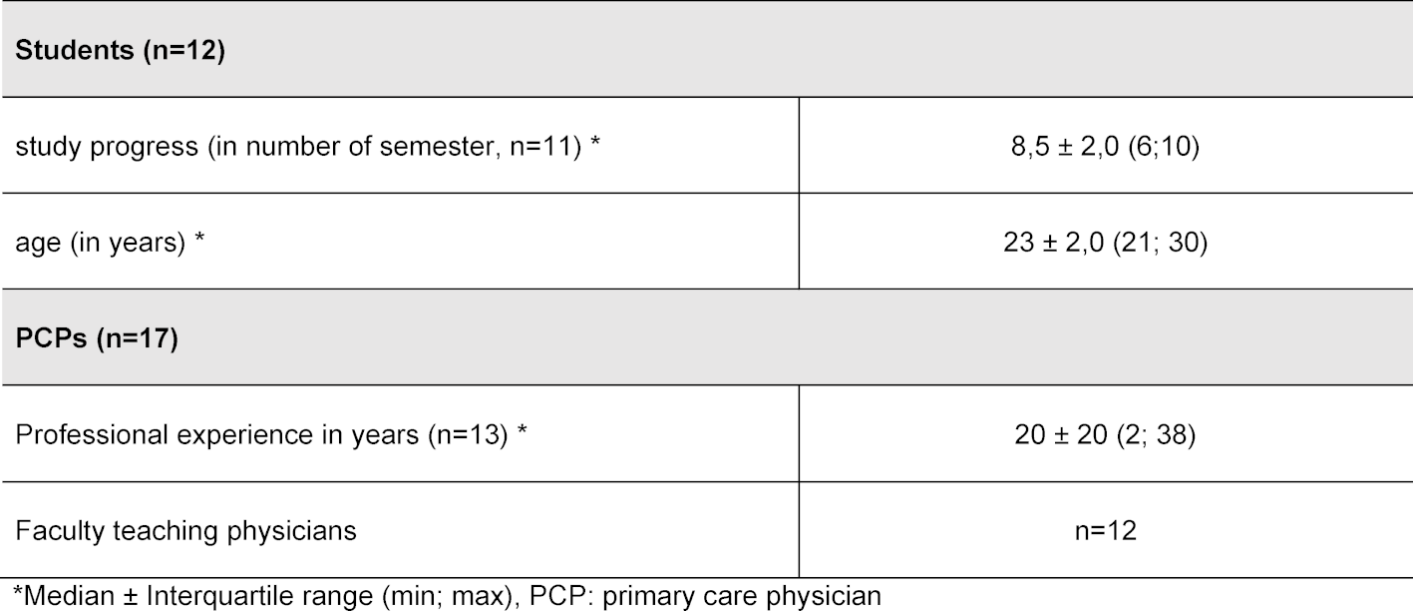
Sociodemographic data and characteristics of the participants interviewed
