# Supplementary material for: The teaching and learning environment of a primary care medical student clinical attachment (“Famulatur”) – a qualitative study on experiences of students and primary care physicians in Germany
Source: GMS J Med Educ. 2019 May 16;36(3):Doc28. doi: 10.3205/zma001236 (PMC6545615; doi:10.3205/zma001236)
Supplement: Content analysis: main categories and selected subcategories from interviews with family physicians (PCP: primary care physician) [file JME-36-3-28-s-001.pdf]

Main category	Subcategory	Typical example
**Associations with Famulatur in primary care**	Students without prior knowledge about primary care	*"Well, the experience with the students is also, 'Yeah, well, he's doing a bit of the typical: cold, hoarseness and cough, but then they're surprised by the diversity, ... that it takes a lot practicing as a PCP [...]" {HA15}*
	Underestimation of primary care as a specialty	*"[...] that they [the students] underestimate the diversity of the specialty. " {HA9}*
	Better supervision than in other Famulaturs	*"And I also have the feeling that the students enjoy it here, because of the 1:1 supervision and this diversity of primary care, they probably don't see that in other specialties." {HA1}*
	Necessity and urgency	*„...[the Famulatur in primary care is] necessary and urgently needed, so that I can increase my activity [as a teacher] and give something back to these young ladies and gentlemen."* {HA10}
**Strengths of the Famulatur in primary care**	Gain insight into primary care	*"Well, it used to be common that you could be an attending physician in internal medicine without ever having seen a primary care practice from inside and without any of the ideas you gain when you experience everyday life in a practice during the Famulatur.* {HA2}
	Get to know challenges	*"What are the problems, the challenges, the possibilities? You don't learn that from any textbook, you just have to experience it. That's the strength of the Famulatur in primary care, even if you don't go into primary care later."* {HA2}

Main category	Subcategory	Typical example
	To inspire students for primary care	*"So the best thing is that people [students] are open and full of expectations and when they get here, they can get excited about getting to know primary care."* {HA7}
	Learn to reflect / to critically question	*"One learns to reflect, for example to critically question specialist findings or hospital discharges notices. You have to put the medication and care into practice in the outpatient setting, which requires a lot of organizational skills, which are demanded and promoted."* {HA9}
	Work independently	*"That the student can work by himself and doesn't just have to walk along."* {HA6}
**Weaknesses of the Famulatur in primary care**	Time pressure	*"I see that as a bit of a problem to accompany the Famulatur with theoretical input. Well, because we already have a tight time schedule and then, for example, to point out certain findings again theoretically or to talk them through again, that's of course not always possible."* {HA16}
	Patient contact harder to establish than in in-patient care	*"Yes, it's just that it's a bit more difficult here than in the hospital, where you can easily get straight into working, straight into patients, so to speak."* {HA8}
	Quality of the Famulatur depends on the supervising physician	*"I think that's up to every colleague. It's always like that, either I'm happy with a Famulatur and ended up in a good practice, or [...] I'm just not."* {HA5}

Main category	Subcategory	Typical example
**Suggestions for improvement**	Introductory and summary discussion between student and supervising physician	*"Yes, introductory discussion, and summary discussion, as you do with the students, in the Blockpraktium."* {HA7}
	Financial compensation for PCPs	*"Well, in itself I think it's good that you teach [...] and of course you can also think about a little compensation for the Famulatur in financial matters, because it's a burden in a way. So you need more time and so on and you don't have that at your disposal, so some kind of compensation would not be bad."* {HA3}
