# Supplementary material for: The teaching and learning environment of a primary care medical student clinical attachment (“Famulatur”) – a qualitative study on experiences of students and primary care physicians in Germany
Source: GMS J Med Educ. 2019 May 16;36(3):Doc28. doi: 10.3205/zma001236 (PMC6545615; doi:10.3205/zma001236)
Supplement: Main and selected subcategories of content from student interviews (PCP: primary care physician) [file JME-36-3-28-s-002.pdf]

Main category	Subcategory	Anchor example
**Associations with Famulatur in primary care**	Primary care as a career choice	*"So of course I immediately think back to my time there [at the practice], I found it very nice, which is also because I got a really positive impression. I was - back then - wondering for the first time whether I should perhaps opt for primary care after all."* {S9}
	Close patient contact	*"Close contact with patients, very personal contact too. Not only in medical issues, but also psychological matters"* {S7}
	Diverse reasons for encounter	*"It was very, very exciting. More interesting than I thought before. [Doctors] were not only concerned about me, but also always [looked] who was doing the most exciting at the practice currently, so I saw a lot of different and really exciting cases."* {S9}
	Chronic Diseases	*"[...] a lot of diabetes and such chronic diseases that you encounter."* {S11}
	„You have to be on your toes."	*"And that you can struggle a bit with the cases and still have to find out for yourself what they [the patients] actually have. And that it [the diagnosis] can be from any specialty. And exactly that there are not only aches and pains, but also real emergencies. And that you have to be on your toes to see that." {S2}*
**Strengths of the Famulatur in primary care**	Optimal supervision	*"I found the 1:1 supervisions very good, and that the doctors also took their time and because everyone [every doctor] had a different focus, I could also get to know quite a lot of different things."* {S6}
	Difference to in-patient care	*"I think it's always good to really get to know a completely different professional life. Here at the university, you live a little like in a bubble [...]." {S3}*

**Weaknesses of the Famulatur in primary care**	Little independent work	*"Maybe the weaknesses is that you're not allowed to do that much yourself [...].“ {S7}*
	Dislike due to obligation	*"I think a potential weakness is that it’s [the Famulatur] mandatory. And I think if you volunteer for something, you'll have more fun." {S10}*
	PCPs are not motivated	*"I don't think every PCP wants it as much as mine does." {S5}*
	Primary care is not a specialty of its own	*"[...] that it is obligatory [...], the Famulatur [is] an excellent opportunity to try out different specializations, and primary care is not a real specialty in itself, but contains all the others somewhere." {S9}*
**Suggestions for improvement**	Guidelines/handbook for PCPs	*"What was missing from the Famulatur, from an organizational point of view was [...] perhaps a list of what should actually be done, perhaps that the doctors could see it as well."* {S3}
	Guidelines/handbook for Students	*"[...] a written list to give to the students of what is expected." {S3}*
	Elimination of Blockpraktikum	*"Exactly, so the Blockpraktikum could be dropped." {S5}*
